# Late Wilate Prophylaxis Initiation in Adult Twins with Type 3 von Willebrand Disease: Overcoming Historical Barriers and Improving Clinical Outcomes

**DOI:** 10.1055/a-2990-4466

**Published:** 2026-11-05

**Authors:** Cristina Sierra, Maria José Ojinaga, Lucia Insunza, Gorka Pinedo, Javier Arzuaga, Juan Carlos García-Ruiz

**Affiliations:** 1 Haemostasis and Thrombosis Unit 16494 Cruces University HospitalBizkaiaBasque CountrySpain; 2 Molecular Biology Laboratory 16494 Cruces University HospitalBizkaiaBasque CountrySpain; 3 Department of Haematology and Haemotherapy 16494 Cruces University HospitalBizkaiaBasque CountrySpain

**Keywords:** Von Willebrand disease, plasma-derived von Willebrand factor, prophylaxis

## Abstract

Prophylaxis with a plasma-derived von Willebrand factor/factor VIII (pdVWF/FVIII) 1:1 concentrate (wilate) is effective in the treatment of severe type 3 von Willebrand disease (VWD). However, uptake of prophylaxis with factor concentrates remains limited due in part to the treatment burden and historical concerns over blood product safety. We describe clinical cases of two adult twin brothers with severe type 3 VWD and a history of hepatitis C acquired from childhood plasma-derived products, who initiated once-weekly pdVWF/FVIII 1:1 concentrate prophylaxis after years of on-demand therapy. Both patients completed 12 months of prophylaxis, which led to a marked improvement in bleeding control and daily functioning, and a decrease in treatment burden compared with on-demand treatment. Their experiences highlight the significant enhancement in quality of life achieved through the adoption of regular prophylaxis. Overcoming long-standing concerns about product safety allowed them to embrace preventive care and realize its benefits in managing their severe VWD.

## Introduction

Von Willebrand disease (VWD) is the most common inherited bleeding disorder, resulting from either a deficiency or dysfunction of von Willebrand factor (VWF). 1 Type 3 VWD is the most severe form and is characterized by a near-total absence of circulating VWF. As VWF normally functions as a carrier protein for coagulation factor VIII (FVIII), individuals with type 3 VWD also exhibit markedly reduced FVIII levels, further contributing to bleeding risk. 1 Clinically, individuals with type 3 VWD exhibit the highest bleeding tendency among the VWD subtypes, although they account for less than 5% of all VWD cases. 1 2

Long-term musculoskeletal complications in individuals with VWD, particularly type 3, arise primarily from recurrent joint bleeding (haemarthrosis), which occurs in approximately half of individuals with type 3 VWD and is strongly linked to very low residual levels of FVIII. 1 3 These recurrent bleeds can lead to arthropathy and reduced joint function, with arthropathy reported in 40% of individuals with VWD following joint bleeds. 1 3 Individuals experiencing more than five joint bleeds in a single joint have a significantly increased risk of developing arthropathy. 3

Current guidelines recommend long-term prophylaxis in individuals with VWD with severe and frequent bleeding episodes (BE) to reduce risk of bleeding and joint damage, and improve quality of life, emphasizing individualised treatment plans based on clinical severity and patient needs. 4 However, prophylaxis use in VWD remains low, compared with, for example, haemophilia A. 5

Historical barriers to prophylaxis in severe VWD include the complexity of identifying and defining severe VWD, diagnostic delays, and underrecognition of the VWD-specific disease burden. 6 Despite guideline recommendations, many adults with severe type 3 VWD remain on on-demand therapy due to historical fears associated with plasma-derived (pd) products. 4 7 8

Prophylaxis in individuals with severe VWD effectively reduces BEs, thus protecting against long-term chronic joint damage. 3 9 Early initiation is beneficial, reducing cumulative morbidity from spontaneous bleeds and trauma-induced haemorrhages. 10 Arthropathy due to recurrent haemarthroses results in chronic pain and reduced mobility and may be less responsive to treatment once chronic damage has occurred. 1 3 Additionally, initiating prophylaxis for VWD later in life may necessitate higher doses and more frequent administration of replacement therapy to control bleeding, thereby increasing the treatment burden and healthcare costs. 5 10 11

The efficacy and safety of prophylaxis with the pdVWF/FVIII 1:1 concentrate (wilate, Octapharma) was demonstrated in adults, adolescents, and children with VWD of all types in the prospective WIL-31 and WIL-33 studies. 9 12 pdVWF/FVIII 1:1 concentrate was well tolerated in all age groups, with no treatment-related serious adverse events, thrombotic events, or treatment-emergent inhibitors reported.

Here, we report the cases of two twin brothers with type 3 VWD who initiated prophylaxis with pdVWF/FVIII 1:1 concentrate later in life, after reluctance due to childhood hepatitis C infection from pd products. These patient cases address a practical challenge that is not fully captured in clinical trials: whether a less burdensome once-weekly start can help reluctant patients experience the benefits of regular prophylaxis, regain confidence in treatment, and transition from on-demand to preventive care.

## Case Presentation

At the time of data cutoff (January 2026), both patients had completed 12 months of prophylaxis. The brothers are currently 49 years old and were diagnosed with type 3 VWD in childhood ( Table 1 ). Laboratory data revealed severely reduced VWF antigen (Patient 1: <2%; Patient 2: 11%) and activity (<4%; 4%) levels and low FVIII coagulant activity (FVIII:C; 1.7%; 2.2%) in both patients, consistent with severe VWD ( Table 2 ). Genetic analysis confirmed type 3 VWD with mutations in exon 52 (c.8347C > T) and intron 137 (c.6598+1G > A), the latter being inherited maternally. Family history includes a deceased sister with the same diagnosis and one sister without VWD; parents had no bleeding history.

**Table 1:** Baseline characteristics.

	Patient 1	Patient 2
Age (y)	49	49
Sex	Male	Male
Body weight (kg)	65	76
Blood type	B−	B−
VWD type	3	3
Genetic mutations	Exon 52 c.8347C>T Intron 137 c.6598+1G>A (Hh)	Exon 52 c.8347C>T Intron 137 c.6598+1G>A (Hh)

Abbreviation: VWD: von Willebrand disease.

**Table 2:** Haemostasis marker and factor levels at baseline, 4, and 7 days after plasma-derived von Willebrand factor/factor VIII 1:1 concentrate infusion.

	Patient 1			Patient 2		
	Baseline	4-d postinfusion	7-d postinfusion	Baseline	4-d postinfusion	7-d postinfusion
Haemostasis						
aPTT (s)	54	36	50	49	43	36
PT (%)	NR	100	96	NR	103	106
Fibrinogen	NR	276	265	NR	302	312
FVIII (%)	1.7	21	1.2	2.2	26	18
VWF/Act (%)	<4	<4	<4	4	<5	<5
VWF/Ag (%)	<2	5	11	11	<8	11

Abbreviations: aPTT, activated partial thromboplastin time; NR, not reported; PT, prothrombin time; FVIII, factor VIII; VWF, von Willebrand factor.

Both patients contracted hepatitis C virus (HCV) in childhood from treatment with plasma derivatives; this infection has since been cured and there is no detectable viral load. No tolerability or safety issues were reported with previous therapies, aside from the HCV infection.

Both patients had a history of joint BEs, leading to chronic damage and pain. Patient 1 exhibited established arthropathy in the right ankle with bone and cartilage alterations and grade II synovial hypertrophy, as well as cartilage and bone alterations in the left ankle and knee ( Fig. 1A ). Patient 2 underwent radiosynoviorthesis in 2014 for chronic synovitis in both ankles. He also had multiple injections with hyaluronic acid and corticosteroids in both ankles and the right knee. Magnetic resonance imaging findings in May 2024 for Patient 2 showed advanced degenerative changes at the tibiofibular-talus joint of both ankles, which extended to the medial and posterior subtalar joint ( Fig. 1B ). Patellofemoral chondropathy was also observed in both knees, with more severity in the left knee where small osteochondral lesions were evident in the external facet of the patella.

**Fig. 1:**
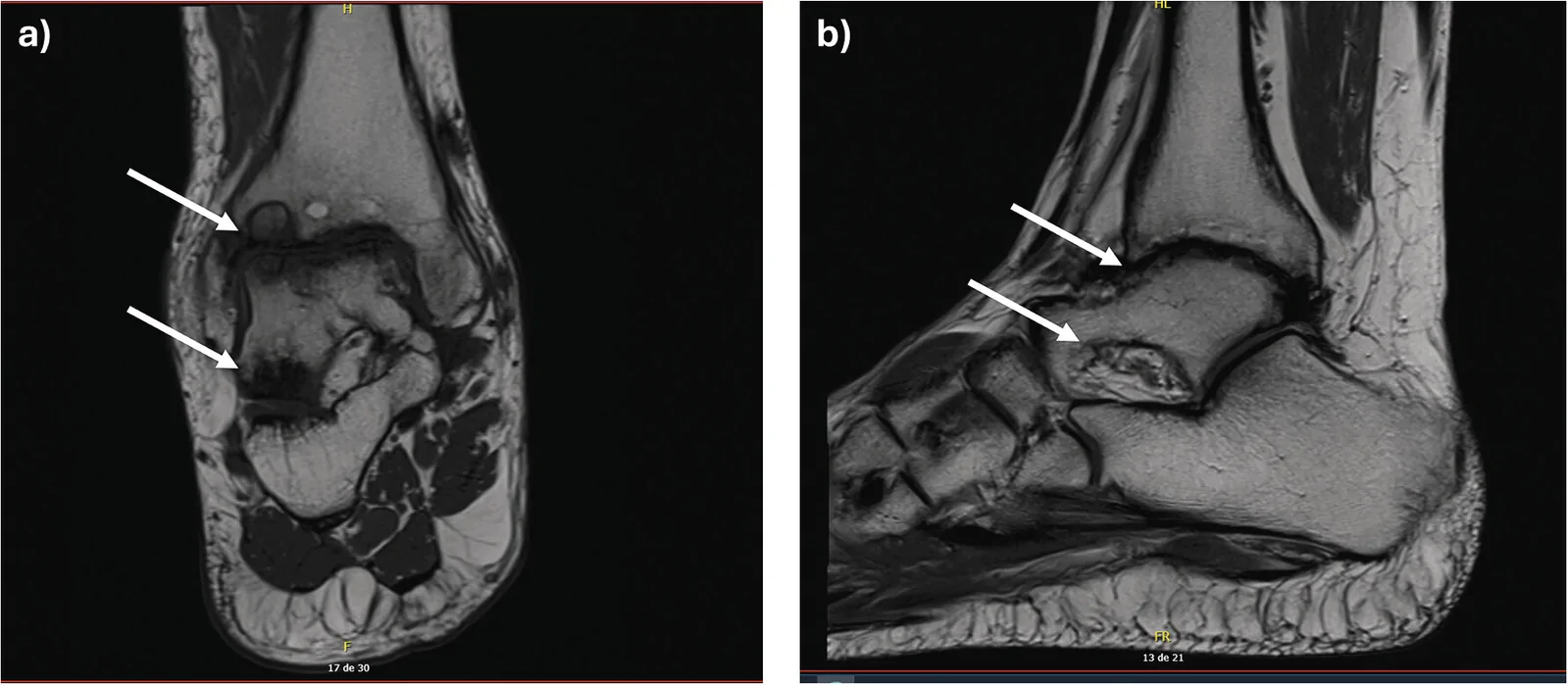
Sagittal magnetic resonance imaging (MRI) of the ankle joint prior to initiation of prophylaxis, demonstrating chronic joint damage in ( **A** ) Patient 1 and ( **B** ) Patient 2.

Both patients were treated on demand with pdVWF/FVIII (Fahndi) prior to initiating prophylaxis with pdVWF/FVIII 1:1 concentrate in January 2025. In the 12 months prior to prophylaxis, Patient 1 had an International Society on Thrombosis and Haemostasis Bleeding Assessment Tool (ISTH-BAT) score of 35, and experienced bleeding symptoms such as epistaxis, cutaneous bleeding, minor wounds, oral cavity bleeding, muscular bleeding, and haemarthrosis ( Fig. 2 ). He visited the emergency room four times due to haemarthrosis in his ankle and knee and reported experiencing pain following increased activity. Severe gingival bleeding necessitated oral iron therapy. Additionally, Patient 1 suffered from mild gastrointestinal bleeding due to ulcerative colitis and occasionally experienced haematuria. In the 12 months prior to prophylaxis, Patient 2 had an ISTH-BAT score of 24, with bleeding that primarily occurred in the gastrointestinal tract, minor wounds, oral cavity, muscles, and joints, including haemarthrosis ( Fig. 2 ). He also experienced frequent nosebleeds and suffered from significant joint pain, necessitating oral iron therapy and imaging for haematuria.

**Fig. 2:**
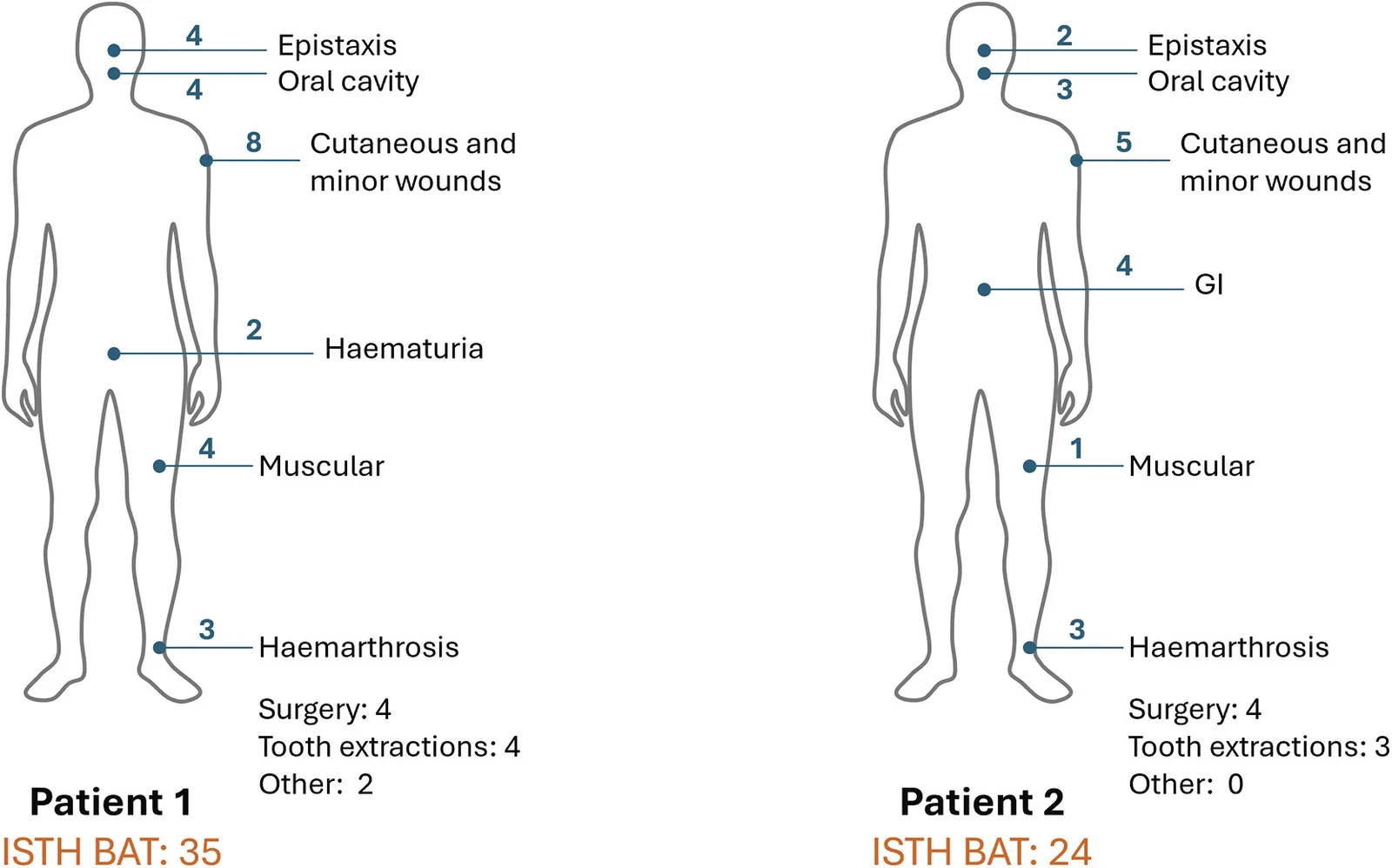
Illustration of haemorrhagic locations and ISTH-BAT score. ISTH-BAT, International Society on Thrombosis and Haemostasis bleeding assessment tool.

Patients were initiated on once-weekly pdVWF/FVIII 1:1 concentrate infusions to help ease acceptance and adherence, with the goal of increasing to twice-weekly infusions once a clear clinical benefit was observed.

Both patients commenced 50 IU/kg once-weekly pdVWF/FVIII 1:1 concentrate prophylaxis in January 2025 (Patient 1: 3,000 IU; Patient 2: 3,500 IU). Prophylaxis was administered in the hospital without any missed doses. The annualised bleeding rate (ABR) was reduced by 91.7% for Patient 1 and by 80% for Patient 2 during once-weekly prophylaxis with pdVWF/FVIII 1:1 concentrate compared with prior on-demand treatment ( Fig. 3 ). No spontaneous breakthrough bleeds have occurred since prophylaxis was initiated. In addition to the substantial improvement in ABR, patients reported clear functional improvement and a reduced impact of VWD symptoms on daily life, easing the overall disease burden. Since initiating prophylaxis, Patient 1 has had no haemarthroses and the gingival bleeding has resolved, and Patient 2 has not experienced further epistaxis. Both patients have also discontinued periodic intravenous iron therapy. In addition, joint pain has resolved and both patients continue to undergo follow-up visits with the rehabilitation team (every 3 and 6 months for Patients 1 and 2, respectively). At this time, no imaging tests have been performed. The patients’ perception of treatment has changed positively, and work is currently underway to intensify the prophylactic regimen to twice weekly.

**Fig. 3:**
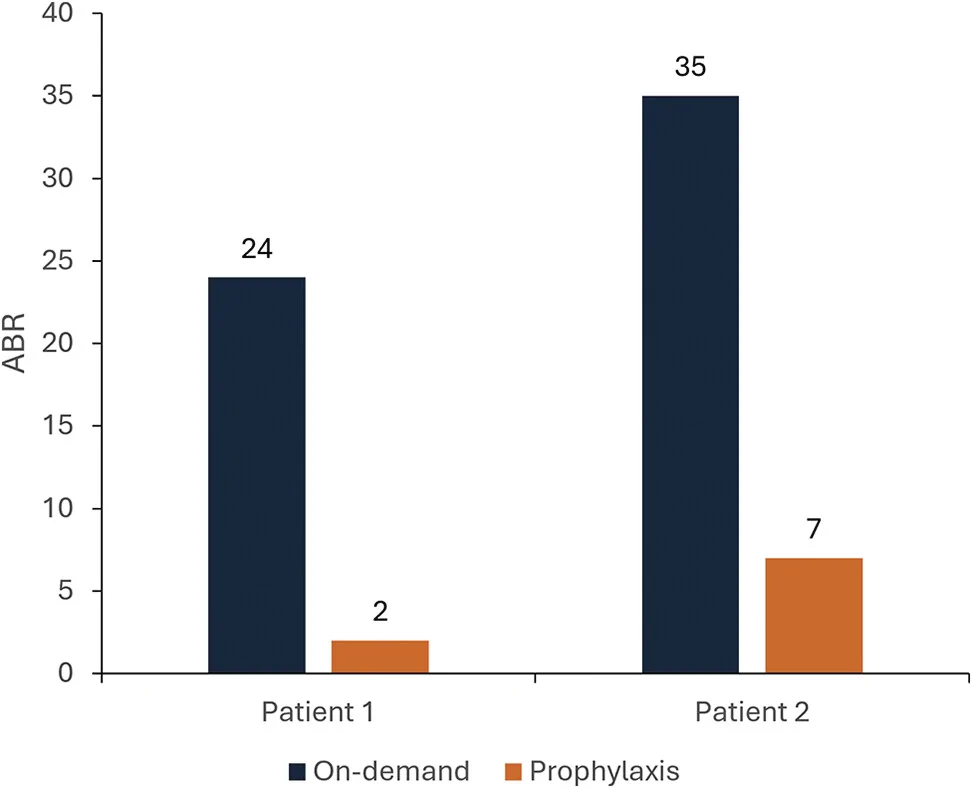
ABR during on-demand treatment and prophylaxis. ABR, annualised bleeding rate.

The weekly prophylaxis schedule was adapted throughout the year to cover minor procedures. For most scheduled procedures, the day of weekly prophylaxis with pdVWF/FVIII 1:1 concentrate was adjusted to match the day of the procedure, with no further treatment required. In the case of Patient 1, an additional pdVWF/FVIII 1:1 concentrate dose was administered 72 h after a dental cleaning and tooth extraction. Patient 2 experienced haematuria following a minor procedure, requiring a second dose of pdVWF/FVIII 1:1 concentrate 72 h later.

At baseline, both patients exhibited very low factor levels. For Patient 2, VWF activity and antigen levels remained unchanged at both 4 and 7 days following infusions with pdVWF/FVIII 1:1 concentrate. In contrast, Patient 1 showed increased VWF antigen levels at both 4 and 7 days’ postinfusion, although there was no change in VWF activity. FVIII levels increased in both patients 4 days after infusion. By 7 days postinfusion, these levels had returned to baseline in Patient 1, while they remained elevated in Patient 2 ( Table 2 ). Both patients showed prolonged baseline activated partial thromboplastin time consistent with severe FVIII deficiency, which was corrected following pdVWF/FVIII 1:1 concentrate infusion, notably with a more sustained improvement in Patient 2. Prothrombin time and fibrinogen remained normal throughout, confirming that coagulopathy was isolated to the VWF/FVIII axis. These haemostasis data demonstrate that pdVWF/FVIII 1:1 concentrate effectively corrected the intrinsic pathway deficit in patients with type 3 VWD, whereas the interpatient variability in FVIII pharmacokinetics suggests that individualised prophylactic dosing may optimise long-term haemostatic cover.

Inhibitor studies were performed monthly, and no treatment-emergent inhibitors were observed. No adverse events or thrombotic events related or unrelated to treatment were reported.

### Patient Perspective

The patient perspectives below highlight that the most meaningful benefit of prophylactic treatment was not only fewer BEs, but also the replacement of unpredictable emergency-driven care with a scheduled, manageable weekly routine.

#### Patient 1

*Before prophylaxis, when I was treated on demand, my life was restricted; you adapt your habits to the risk of bleeding and live with joint pain. My main concerns about prophylaxis were safety, frequency and being able to fit infusions around my work schedule. As my symptoms worsened, I started looking for alternatives and agreed to prophylaxis. After starting prophylaxis, bleeding decreased and the sense of safety increased. A scheduled weekly infusion is easier to manage than unexpected bleeds. The main burden of prophylaxis is fitting it into my daily schedule, as I have it administered at the hospital. However, it has become part of my routine and is the ideal treatment compared to on-demand. The benefits of prophylaxis considerably outweigh the inconvenience* .

#### Patient 2

*Before starting prophylaxis, the main complication I experienced was haemarthrosis, which significantly limited my mobility due to the pain it caused. My main concern about prophylaxis was the safety of the products used, given my previous bad experience with associated infections from contaminated blood products. Without a doubt the persistence of my haematologist (Dr. Sierra) and the deterioration of my mobility due to the existing chronic pain were the main reasons I agreed to prophylaxis. What has changed the most since starting prophylaxis, has been the reduction in gum bleeding and joint haemorrhages, which has significantly improved my quality of life in every way. A scheduled weekly prophylactic infusion is much simpler and more effective than any emergency or on-demand treatment, and given the reduction in symptoms, the benefits far outweigh any associated inconvenience. A weekly infusion involves minimal burden in all aspects, as long as the medication is available at the scheduled time; I have integrated it into my weekly routine without major complications. To other patients with severe VWD hesitant about prophylaxis, I would say that their quality of life will improve significantly with minimal effort.*

## Discussion

We present the cases of twin brothers with type 3 VWD who experienced a significant reduction in disease burden after initiating once-weekly prophylaxis with pdVWF/FVIII 1:1 concentrate despite initial reluctance. Both brothers experienced a substantial bleeding burden and joint morbidity prior to prophylaxis, with recurrent haemarthrosis leading to arthropathy and functional limitations, consistent with data from multiple studies showing high rates of joint damage in VWD patients with frequent bleeding. 1 3

Hepatitis C acquired from blood products in childhood contributed to their reluctance to initiate the regular infusions required by long-term prophylaxis. The once-weekly prophylaxis used in these cases should not be interpreted as a substitute for standard prophylaxis regimens but rather as a pragmatic and patient-centered approach to reduce psychological and logistical barriers in patients with longstanding reluctance to regular prophylaxis, with the planned goal of titrating up to twice-weekly if required.

Late initiation of prophylaxis in individuals with type 3 VWD significantly reduces bleeding rates and prevents further joint damage, even after longstanding bleeding and established arthropathy. 5 The WIL-31 study found an 84.4% reduction in mean total ABR (TABR) and a 93% decrease in total joint bleeds following the switch from on-demand treatment to prophylaxis with pdVWF/FVIII 1:1 concentrate across all VWD types. 9 13 In individuals with type 3 VWD, TABR was reduced by 86%, spontaneous ABR by 89%, and overall mean joint ABR by 92.4%. 13 Although existing joint damage cannot be reversed, prophylaxis preserves remaining joint function and improves quality of life by reducing recurrent bleeds and associated morbidity. 5 14 This approach mirrors the rationale in severe haemophilia, emphasizing long-term bleed prevention to mitigate chronic complications and enhance patient outcomes. 5 9

In the cases described here, once-weekly pdVWF/FVIII 1:1 concentrate infusions reduced ABR and improved daily functioning compared with on-demand treatment. It was also practical and safe, with no adverse events or inhibitor development reported. Intensifying the prophylaxis regimen to twice-weekly dosing may provide enhanced bleed prevention and better control of breakthrough bleeding in individuals needing more robust protection. 13 This stepwise approach allows for personalised treatment adjustments based on clinical response and patient tolerance and can aid in overcoming barriers such as patient hesitation and inhibitor management. 14 It can provide a practical method to optimise individual patient preferences and outcomes. 13 14 Moreover, both patients described a shift from living with the burden of bleeding risk to living with greater predictability and safety. This patient-reported improvement in quality of life is central to the value of prophylaxis, particularly in adults with a longstanding reluctance to initiate regular prophylaxis treatment.

Educating patients on the benefits of prophylaxis, including improved quality of life and prevention of long-term joint damage, supports adherence and informed shared decision-making between patients and clinicians. 5 8 15 Enhanced patient–physician communication and shared decision-making further support personalised management approaches that address both clinical efficacy and patient concerns.

## Conclusion

In our experience, even a less intensive once-weekly pdVWF/FVIII 1:1 concentrate prophylaxis regimen decreased bleeding frequency and improved the daily lives of twin brothers with type 3 VWD, while also potentially preventing long-term issues such as arthropathy. These two cases underscore the potential for clinical benefit by transitioning from on-demand treatment to regular prophylaxis via a stepwise approach in patients with severe type 3 VWD. This highlights the efficacy, safety, and flexibility of pdVWF/FVIII 1:1 concentrate for personalised management in severe VWD.

## Declaration of GenAI Use

Paperpal (AI-assisted writing tool) was used for drafting only; all content was reviewed, validated, and approved by the authors. The authors had full control over the content and decision to publish.
